# A Case of Hæmatic Filariasis in a Dog

**Published:** 1902-08

**Authors:** Charles F. Dawson

**Affiliations:** Lake City, Fla.


### REPORTS OF CASES.

### A CASE OF HÆMATIC FILARIASIS IN A DOG.

By Charles F. Dawson, V.S.,
LAKE CITY, FLA.

Six months ago a hunting dog was brought to me suffering, so the owner stated, with rheumatism. The animal had been treated through the "Veterinary Column" of a newspaper unsuccessfully. I made a careful examination, but failed to find any soreness of the muscles or joints. The hind legs appeared quite slender, and shifting lameness was a prominent symptom. There was shortness of breath, slight fever, and a somewhat tumultuous heart, although no murmur could be detected with the unaided ear. The appetite was good and thirst pronounced. Emaciation was somewhat marked, and the dog was nervous and debilitated. The anus was relaxed, and the breath was foul and the eyes sunken. Suspecting heart disease, I prescribed a heart tonic, which benefited the animal. The owner finally abandoned treatment and the case passed from my notice. Finally the owner notified me the animal was down, in convulsions, and that he expected it to die at any moment. The animal died during my absence, and, upon my return two days later, I exhumed the body and made an examination. Decomposition had progressed to a stage which precluded a minute description of the changes which must have taken place in the important viscera.

There was considerable bloody effusion into the peritoneal, pleural, and pericardial cavities. Upon section of the heart a mass of worms appeared in the right ventricle. They were twisted in every conceivable direction around the columnæ carnæ. Study showed the worms to be filaria immitis. There were four females, the largest being 30 centimetres long, and two males, each about 18 centimetres in length.

Reports of the sudden deaths of dogs lead me to believe that this affection is common in Florida.

---

Dr. O. M. Norton, a graduate of the University of Pennsylvania, class of 1901, has succeeded Dr. H. M. Watson, who has located at Haverhill, Mass.

